# Cross-Sectional Analysis of Oral Healthcare vs. General Healthcare Utilization in Five Low- and Middle-Income Countries

**DOI:** 10.3389/froh.2022.911110

**Published:** 2022-06-23

**Authors:** Sita Manasa Susarla, Margaret Trimble, Karen Sokal-Gutierrez

**Affiliations:** School of Public Health, University of California, Berkeley, Berkeley, CA, United States

**Keywords:** oral health, dental care, prenatal care, child immunizations, LMICs, dental caries, healthcare, maternal-child health

## Abstract

Oral health is integral to overall health and is often neglected, especially in low- and middle-income countries (LMICs). Oral disease, including untreated dental caries, affects nearly 3.5 billion people globally, contributing to poor health and quality of life. To examine the relationship between the utilization of general healthcare and oral healthcare, we conducted an exploratory cross-sectional study of first-visit interview data collected from a convenience sample of 3,422 low-income mothers and 4,324 children aged 6 months through 6 years participating in a community-based oral health and nutrition program in five LMICs (Ecuador, El Salvador, India, Nepal, and Vietnam) from 2006–2015. We used descriptive and exploratory association analysis to identify patterns of oral healthcare utilization for mothers and children compared to medical care utilization, specifically maternal prenatal care and child immunizations. Overall, 89.6% of the mothers had received prenatal care for at least one child, but only 76.4% had ever received dental care and 50% were currently suffering from oral health symptoms, primarily oral pain. Mothers who received prenatal care were significantly more likely to have accessed dental care compared to those who had not received prenatal care (OR = 2.62, 95% CI: 2.06, 3.32). Overall, 95.4% of the children had current immunizations, but only 30.1% had ever received dental care, and 32.4% were currently suffering from oral pain. Children whose immunizations were up-to-date were more likely to have received dental care, with a significant association in Ecuador (OR = 3.29, 95% CI: 2.06, 5.30). Compared to utilization of general healthcare, oral healthcare was under-utilized by mothers and children in our sample from five LMICs. Integration of prevention- and treatment-oriented oral healthcare into primary medical care services, particularly prenatal care and child immunizations, could help increase access to oral healthcare and improve women's and children's oral health.

## Introduction

Oral health is intimately linked with general health and has physiological, psychological, and social ramifications that affect well-being [1]. Oral disease, which includes dental caries and periodontal disease, affects nearly 3.5 billion people globally, making it the most prevalent non-communicable disease (NCD) [2]. Dental caries is the most common chronic childhood disease, affecting between 60 and 90% of children worldwide, and untreated dental caries in permanent teeth affects 35% of the global population [3]. Oral health also has a bidirectional relationship with other NCDs [4], such as cardiovascular disease and diabetes, through complex physiological interactions as well as shared risk factors such as tobacco use and high sugar consumption [5–8]. Nevertheless, oral health is often neglected, and oral healthcare is accessed differently from general healthcare, particularly in low- and middle-income countries (LMICs). In many LMICs, most carious lesions remain untreated due to inappropriate, unaffordable or unavailable oral healthcare services, contributing to poor overall health and quality of life [9].

Despite the fact that dental caries and periodontal disease are largely preventable, improvements in oral health and oral healthcare access have remained challenging, especially in LMICs [10–13]. Oral healthcare access ranges from 35% in low-income countries to 75% in upper-middle income countries [14]. Low oral healthcare access is driven in part by the routine omission of oral healthcare from Universal Health Coverage [15], as well as the biomedical model of dentistry that incentivizes treatment-oriented oral healthcare, including fillings, crowns, and extractions, over prevention-oriented oral healthcare such as routine checkups, cleanings, application of fluoride varnish, and oral hygiene instruction [16, 17]. This traditional model perpetuates the demand for highly-skilled dentists; however, in many LMICs, insufficient resources have been allotted to cultivate a prevention-oriented oral healthcare workforce and approach [10, 18]. Finally, the rise in the overconsumption of sugary food products, specifically sugar-sweetened beverages, has exacerbated oral disease and has contributed greatly to the lack of improvement in global oral health [19, 20].

In contrast, numerous improvements have been made in maternal and child health in recent decades. The estimated worldwide coverage of early prenatal care visits increased by 43.3%, from 40.9% in 1990 to 58.6% in 2013 [21]. According to UNICEF, global data show that 87% of pregnant women access prenatal care at least once during their pregnancy, and 59% receive four or more prenatal care visits [22]. Similarly, progress has been made in immunization coverage throughout the world since the establishment of the Expanded Programme on Immunization by the World Health Organization (WHO) in 1974. According to the WHO, despite disruption caused by the COVID-19 pandemic, 83% of infants worldwide received three doses of diphtheria-tetanus-pertussis vaccine in 2020 [23]. Altogether, these developments have improved accessibility to preventive medical care for mothers and children globally.

The rising prevalence of NCDs in LMICs, including oral disease, threatens the sustainability of Universal Health Coverage [24, 25]. Furthermore, the COVID-19 pandemic has highlighted the increasing need to maintain proper oral health and oral hygiene, such as regular dental visits, as poor oral health has been shown to exacerbate the severity of COVID-19 significantly [26]. Nevertheless, the consensus that oral health is integral to general health has not translated to widespread integration of these two health care domains. Understanding the relationship between the utilization of general preventive healthcare services and dental care, including both treatment- and prevention-oriented oral healthcare services, may illuminate opportunities to expand delivery of oral healthcare and improve overall population health. This cross-sectional study explores dental care and general preventive healthcare utilization in five LMICs (Ecuador, El Salvador, India, Nepal, and Vietnam) and identifies correlations between maternal prenatal care and child immunization access, and dental care utilization.

## Materials and Methods

### Ethical Approval

This family of studies on children's oral health was developed and conducted as a collaboration among University of California Berkeley with local country Ministries of Health, academic institutions, and non-governmental non-profit organizations. All study sites and protocols were approved by the Committee for the Protection of Human Subjects, the Institutional Review Board (IRB) at the University of California, Berkeley (#2010-06-1655, #2011-04-3176, #2011-04-3178, and #2012-11-4798). Additional approval was obtained, when required, by IRB reliance from the University of California, San Francisco, local ministries of health (El Salvador, Ecuador), health research council (Nepal), university (Vietnam), and non-profit organization directors (El Salvador, Ecuador, Vietnam, Nepal, and India).

### Study Design and Population

This is a cross-sectional descriptive study of a convenience sample of 3,422 low-income mothers and 4,324 children aged 6 months through 6 years who participated in a community-based program promoting child nutrition and oral health in two LMICs in Latin America (Ecuador and El Salvador) and three LMICs in Asia (India, Nepal, and Vietnam). The communities were rural (Ecuador, El Salvador, Nepal), urban (India, Nepal, Vietnam), and peri-urban (Vietnam). The local partner organizations identified study communities where they worked, and all mothers and children in the study age group were invited to participate. This paper presents data collected from 2006–2015: Ecuador from 2010–2013, El Salvador from 2006–2010 and 2014, Nepal and Vietnam from 2011–2013, and India from 2012–2015. For this analysis, we included only the first visits of mothers with the children present on that first visit. We excluded data from caregivers who were not the mothers of the children, and children outside the age range criteria to focus on the ages when primary teeth comprise the majority of a child's dentition [27].

### Data Collection

In each site, trained local health workers provided mothers with a verbal explanation of the study in their native language, and obtained written/verbal informed consent for the mother's and child's participation. The consent forms and the surveys were validated through forward and back translation and pilot samples to confirm understanding of the questionnaire. Results using the questionnaires have been previously published [13, 28–31]. The survey was modified from the WHO oral health survey [32] and consisted of 49 questions: 20 questions regarding maternal background, nutrition and oral health history and habits, access to general healthcare and oral healthcare, and knowledge and beliefs about oral health and disease; and 29 questions about each child's nutrition and oral health history and habits, access to general healthcare and oral healthcare, and oral health status. In El Salvador, Ecuador, Nepal and India, trained volunteers and community health workers fluent in the local/native languages interviewed the mothers and completed both sections of the survey; and in Vietnam, the partner organization requested that the survey be completed directly by mothers since their education and literacy levels were high. Responses were recorded on paper forms before being entered into an Excel spreadsheet or immediately entered on computer tablets into a Qualtrics database.

### Statistical Analysis

The entered data were cleaned and analyzed via RStudio (version 4.0.3, RStudio PBC, Boston, MA, USA) and Microsoft Excel (version 2201, Microsoft Corporation, Albuquerque, NM, USA). Missing data that could not be imputed were ignored in the final analysis. In our univariate analysis, we first calculated descriptive statistics such as averages, percentages, and ratios to identify patterns of oral healthcare utilization compared to medical care utilization among mothers and children. We then used Fisher's Exact Test, a procedure used for small sample sizes with one or more cells less than an expected value of 5, to calculate odds ratios and 95% confidence intervals to further estimate the effect size and confirm the directionality of the relationship between the utilization of any kind of dental care and preventive medical care, specifically maternal prenatal care and child immunizations. Where appropriate, we used the delta method to calculate the 95% confidence intervals for the ratios. Statistical significance was considered for *p*-values < 0.05.

## Results

### Mother and Child Demographic Characteristics

A total of 3,422 mothers and 4,324 children aged 6 months through 6 years were studied. Across all five countries, the mothers had a mean age of 29 years and had 2.3 children. Overall, mothers obtained roughly 7 years of education, and half of mothers (48.8%) had 8 years of schooling or more, one-third (35.2%) had some primary school level of education, and 1 in 6 (16.0%) had no formal education. The children had a mean age of 3.7 years, and a slightly greater proportion were male (52.9%) than female (47.1%) (Table 1).

**Table 1:** Mother and child demographics.

	**Ecuador** ***N*** **= 553** **mothers** ***N*** **= 869** **children**	**El Salvador** ***N*** **= 691** **mothers** ***N*** **= 829** **children**	**India** ***N*** **= 691** **mothers** ***N*** **= 945** **children**	**Nepal** ***N*** **= 863** **mothers** ***N*** **= 1,057** **children**	**Vietnam** ***N*** **= 624** **mothers** ***N*** **= 624** **children**	**Total** ***N*** **= 3,422** **mothers** ***N*** **= 4,324 children**
**Maternal Characteristics:**						
Mean maternal age (years)	29.7 ± 8.1	28.3 ± 7.4	26.8 ± 4.9	28.6 ± 5.6	32.9 ± 5.0	29.1 ± 6.5
Mean number of children per mother	3.5 ± 2.4	2.4 ± 1.6	2.2 ± 1.1	2.0 ± 1.1	1.6 ± 0.8	2.3 ± 1.6
Maternal education, mean years completed	8.3 ± 4.3	5.7 ± 3.7	6.1 ± 4.0	5.6 ± 4.3	13.0 ± 3.7	7.4 ± 4.8
**Education Completed by Mother (%)**						
0 Years	5.3	12.1	14.7	27.9	14.5	16.0
1–7 Years	41.2	56.4	43.3	29.5	6.4	35.2
8+ Years	53.5	31.5	42.0	42.5	79.1	48.8
**Child Characteristics:**						
Mean child age (years)	3.5 ± 1.8	3.6 ± 1.8	3.5 ± 1.8	3.8 ± 1.7	4.1 ± 0.9	3.7 ± 1.7
**Child Sex (%)**
Male	53.4	49.9	54.5	54.1	52.2	52.9
Female	46.6	50.1	45.5	45.9	47.8	47.1

### Mothers' Access/Utilization of Dental Care vs. General Healthcare

Across all five countries, nearly all mothers (95.8%) reported ever visiting the doctor (Table 2). Moreover, 9 out of 10 mothers (89.6%) had received prenatal care for at least one child present with her at the time of the interview, ranging from 79% in the India sample to 100% in the Vietnam sample. Overall, mothers reported an average of 7.8 prenatal care visits, ranging from roughly 6 in the Nepal sample to 9 in the El Salvador sample.

**Table 2:** Mothers' utilization of general healthcare and dental care.

	**Ecuador** ***N*** **= 553** **mothers**	**El Salvador** ***N*** **= 691** **mothers**	**India** ***N*** **= 691** **mothers**	**Nepal** ***N*** **= 863** **mothers**	**Vietnam** ***N*** **= 624** **mothers**	**Total** ***N*** **= 3,422** **mothers**
**General Healthcare:**						
Mothers who have ever visited the doctor (%)	92.2	93.4	96.8	96.5	99.5	95.8
Mothers who received prenatal care for at least 1 child (%)	87.0	95.5	79.2	87.7	100.0	89.6
Average number of prenatal care visits per mother	7.9 ± 5.9	9.0 ± 3.7	8.7 ± 6.7	6.3 ± 3.3	7.2 ± 2.9	7.8 ± 4.7
**Dental Care:**						
Percentage of mothers who have ever visited the dentist (%)	89.9	77.6	56.1	67.9	97.9	76.4
Percentage of mothers who have never visited the dentist (%)	10.1	22.4	43.9	32.1	2.1	23.6
Average number of months since last dental visit	7.8 ± 12.8	17.7 ± 27.3	22.5 ± 27.0	22.6 ± 24.8	10.0 ± 14.3	14.8 ± 22.6
Ratio of any doctor visits to any dental visits	1.03 (0.99–1.06)	1.20 (1.15–1.26)	1.73 (1.61–1.85)	1.42 (1.35–1.49)	1.02 (1.00–1.03)	1.25 (1.23–1.28)
Ratio of any prenatal care visits to any dental visits	0.97 (0.93–1.01)	1.23 (1.18–1.29)	1.41 (1.31–1.52)	1.29 (1.23–1.36)	1.02 (1.01–1.03)	1.17 (1.15–1.20)
Reasons for Last Dental Visit (%)^*^						
Symptoms and treatment only:	67.0	70.4	96.2	88.3	74.9	76.6
Pain	34.6	41.1	65.7	49.0	32.5	42.5
Bleeding gums	1.2	2.8	11.9	12.5	2.9	5.1
Tooth/molar decay	8.2	23.0	15.7	12.5	21.1	16.7
Tooth/molar filling	23.0	22.6	22.6	5.8	3.4	16.5
Tooth/molar extraction	18.2	29.0	5.4	22.9	6.1	17.3
Prevention only: (checkup and/or cleaning)	33.0	29.6	3.8	11.7	25.1	23.4
Ratio of treatment to preventive dental care	2.03	2.38	25.58	7.55	2.98	3.27
Recent Oral Health Concerns (%)^*^						
At least 1 oral health concern	58.4	51.9	47.8	29.3	80.3	50.3
Pain	45.4	38.5	37.6	16.7	33.4	32.8
Rotten tooth/molar	17.2	21.1	3.8	5.8	13.3	11.9
Bleeding gums	5.8	16.1	9.3	10.8	12.7	11.1
Inflammation	6.7	9.6	2.2	1.6	20.0	7.1
Other	0.9	–	2.4	3.1	5.6	2.9

* *Survey questionnaire allowed selection of multiple reasons for dental visit and recent oral health concerns within the last 3 months. Therefore, values may not add up to 100%*.

Overall, only three-quarters of mothers (76.4%) reported receiving any kind of dental care, with the lowest utilization (56.1%) occurring in the India sample. Across all five countries, mothers were 1.3 times (or 25%) more likely to have gone to a doctor for any reason than to have received any kind of dental care (95% CI: 1.23, 1.28), and 1.2 times (or 17%) more likely to have received prenatal care than to have received any kind of dental care (95% CI: 1.15, 1.20). The average time since the last dental care visit of any kind was 14.8 months, ranging from 7.8 months in the Ecuador sample to 23 months in the India and Nepal samples. Overall, dental visits that were driven by oral symptoms or were treatment-oriented were 3 times more common than those that were exclusively prevention-oriented, and the difference ranged from 2- to 3-fold in the Ecuador, El Salvador and Vietnam samples to 8-fold in the Nepal sample and 26-fold in the India sample. Overall, and in each country, dental pain was the most common symptom cited as motivating the last dental visit.

Half of mothers overall (50.3%) reported experiencing recent dental symptoms, ranging from 29% in the Nepal sample to 80% in the Vietnam sample. Dental pain was the most common symptom, reported by one-third (32.8%) of all mothers, ranging from 17% in the Nepal sample to 45% in the Ecuador sample.

Receiving prenatal care was significantly associated with the utilization of any kind of dental care services (OR = 2.62; 95% CI: 2.06, 3.32). This association was statistically significant in all countries except India (OR = 1.16; 95% CI: 0.79, 1.71) and Vietnam (for which the OR could not be calculated since 100% of mothers reported receiving prenatal care for at least one child) (**Table 4**).

### Children's Access/Utilization of Dental Care vs. General Healthcare

Across all five countries, nearly all children (95.4%) were reported to have up-to-date immunizations (Table 3). However, less than a third of children (30.1%) had ever visited a dentist. The average age of the children who had visited the dentist was 4.3 years and these children averaged 3 dental visits. Across all countries, 70% of children had never received any kind of dental care, ranging from 34% in the Ecuador sample to 86-87% in the Nepal and India samples. Across all five countries, children were over 3 times more likely to have been up-to-date with their immunizations than to have received any kind of dental care (95% CI: 3.02, 3.32), and the difference ranged up to 7- to 8-fold in the Nepal and India samples.

**Table 3:** Children's utilization of general healthcare and dental care.

	**Ecuador** ***N*** **= 869** **children**	**El Salvador** ***N*** **= 829** **children**	**India** ***N*** **= 945** **children**	**Nepal** ***N*** **= 1,057** **children**	**Vietnam** ***N*** **= 624** **children**	**Total** ***N*** **= 4,324** **children**
**General Healthcare:**						
Children whose immunizations are up-to-date (%)	88.7	96.7	97.1	97.2	97.5	95.4
**Dental Care:**						
Children who have ever visited the dentist (%)	66.0	21.0	12.7	13.8	50.9	30.1
Children who have never visited the dentist (%)	34.0	79.0	87.3	86.2	49.1	69.9
Average age among those who went to the dentist	4.0 ± 1.7	4.8 ± 1.6	5.0 ± 1.5	4.3 ± 1.6	4.3 ± 0.9	4.3 ± 1.5
Average number of dental visits among those who visited the dentist	3.4 ± 5.1	3.1 ± 5.6	2.0 ± 1.4	2.3 ± 1.8	2.8 ± 1.9	3.1 ± 4.4
Ratio of up-to-date immunization visits to any dental visits	1.34 (1.27–1.42)	4.60 (4.03–5.26)	7.66 (6.45–9.09)	7.04 (6.05–8.20)	1.92 (1.76–2.09)	3.17 (3.02–3.32)
**Reason for Last Dental Visit**						
Symptoms and treatment only: (caries, pain, filling, extraction)	44.5	50.9	87.5	69.4	64.1	55.0
Prevention only: (checkup, cleaning, fluoride treatments, oral hygiene)	55.5	49.1	12.5	30.6	35.9	45.0
Ratio of treatment to preventive dental care	0.80	1.04	7.00	2.27	1.78	1.22
**Current Oral Health Concerns**						
Any report of oral pain (%)	40.4	25.6	32.8	17.4	62.1	32.4
Oral pain interfering with eating (%)	28.4	16.7	23.6	13.6	41.8	23.2
Oral pain interfering with sleeping (%)	23.4	8.0	20.0	9.4	22.7	16.0
Mother's Opinion that Child's Oral Health is Poor (%)	14.4	24.5	20.9	16.8	16.2	18.6
Mother's opinion that child's overall health is poor (%)	6.1	20.9	15.2	4.6	2.2	9.9
Ratio of poorly rated oral health to overall health	2.37 (1.73–3.25)	1.17 (0.98–1.40)	1.37 (1.11–1.69)	3.66 (2.69–4.98)	7.40 (4.19–13.05)	1.87 (1.67–2.09)

Overall, of the 30.1% of children who had ever visited a dentist, over half (55%) of the visits were driven by oral symptoms or were treatment-oriented whether in conjunction with a prevention-oriented service or not, while less than half (45%) of visits consisted exclusively of prevention-oriented services such as checkups, cleanings, application of fluoride treatments, and oral hygiene education. Treatment-oriented dental visits were 1.2-times more common than those that were exclusively prevention-oriented, and the difference ranged up to 7-fold in the India sample. Overall, one-third (32.4%) of children were reported to currently complain of oral pain, ranging from 17% in the Nepal sample to 62% in the Vietnam sample. Furthermore, 1 in 4 children (23.2%) were reported to have oral pain that interfered with eating, and 1 in 6 children (16%) to have oral pain that interfered with sleeping.

Mothers consistently rated their child's oral health as inferior to their child's overall health. Collectively, a designation of “poor oral health” was nearly twice as common as that of “poor overall health,” with differences ranging to nearly 4-fold in the Nepal sample to over 7-fold in the Vietnam sample.

Being current with immunizations was significantly associated with utilization of any kind of dental care services only in Ecuador (OR = 3.29; 95% CI: 2.06, 5.30). This association was not significant overall, likely due to the low proportion of children who had ever seen a dentist (OR = 1.18; 95% CI: 0.84, 1.69) (Table 4).

**Table 4:** Maternal-child access to dental care associated with prenatal care and immunizations.

	**Ecuador** ***N*** **= 553** **mothers** ***N*** **= 869** **children**	**El Salvador** ***N*** **= 691** **mothers** ***N*** **= 829** **children**	**India** ***N*** **= 691** **mothers** ***N*** **= 945** **children**	**Nepal** ***N*** **= 863** **mothers** ***N*** **= 1,057 children**	**Vietnam** ***N*** **= 624** **mothers** ***N*** **= 624** **children**	**Total** ***N*** **= 3,422** **mothers** ***N*** **= 4,324** **children**
**Mothers:**						
Odds ratio^a^ (95% CI)	2.54^†^ (1.17–5.23)	4.63^**^ (2.09–10.42)	1.16 (0.79–1.71)	1.95^**^ (1.26–3.01)	–	2.62^**^ (2.06–3.32)
P-value	0.01	<0.01	0.45	<0.01	–	<0.01
**Children:**						
Odds ratio^b^ (95% CI)	3.29^**^ (2.06–5.30)	1.50 (0.50–6.09)	2.70 (0.42–113.00)	4.62 (0.75–190.46)	1.02 (0.27–3.86)	1.18 (0.84–1.69)
*P*-value	<0.01	0.63	0.50	0.17	1.00	0.36

† *p < 0.05;*

** *p < 0.01;*

*CI, Confidence Interval*.

a *The odds of having dental care among women who had prenatal care compared to women who did not have prenatal care*.

b *The odds of having dental care among children whose immunizations were up-to-date compared to children whose immunizations were not*.

## Discussion

This cross-sectional study of a convenience sample of mothers and children from five LMICs (Ecuador, El Salvador, India, Nepal, and Vietnam) described the disparity between maternal-child utilization of general healthcare and dental care services, and explored the relationship between access to preventive medical care and dental care. Mothers, and especially children, had greater utilization of preventive medical care services than dental care services of any kind, including both treatment- and prevention-oriented services, and mothers and children who accessed preventive medical care services had a greater likelihood of utilizing any kind of dental care services.

Mothers primarily sought dental care services for themselves and their children to treat oral pain, commonly from severely-decayed teeth. Collectively, one-third of the mothers had experienced oral pain within the last 3 months and one-third of the children were experiencing oral pain at the time of the interview. Because dental care is often utilized only when symptoms arise, treatment is frequently limited to emergency care such as dental extraction, a common practice in many LMICs [33–35]. Antibiotics are also commonly given to treat abscesses, which can contribute to the proliferation of antibiotic-resistant bacterial strains [36], and analgesics including opioids are commonly given to treat pain, which can contribute to opioid addiction and overdoses [37]. Furthermore, temporary palliative dental treatment, as opposed to definitive care, contributes to the persistence of chronic dental symptoms [38–40]. This misutilization of oral healthcare has been attributed in part to prohibitive costs of care, prompting the need to restructure the traditional model of dentistry and the delivery of oral healthcare services [15, 41, 42].

Few studies have specifically compared the utilization of prenatal care services to the utilization of dental care services. Our exploratory study estimated that mothers were 1.2 times more likely to have ever received prenatal care compared to any kind of dental care (including both treatment-oriented and exclusively prevention-oriented visits). This estimate is consistent with other studies in Sudan and the Solomon Islands which found greater odds of utilization of dental care services given participation in preventive measures such as health screenings [43, 44]. Our study also estimated that children were over 3 times more likely to be up-to-date with immunizations than to have received any kind of dental care. Previous studies by Chi et al. and Tiwari et al. showed that children who received well-child checkups were more likely to access preventive dental care, and that a greater number of well-child checkups was associated with earlier preventive dental care visits among children [45, 46].

A growing body of evidence demonstrates that increasing the accessibility and utilization of oral healthcare for pregnant women and children improves oral health outcomes for both. Studies in Saudi Arabia found that women who visited the dentist regularly had increased oral health knowledge about methods to prevent caries in children [47, 48]. A 2-year randomized controlled trial that examined the impact of an oral health promotion program among Australian Aboriginal pregnant women and their children found that children in the intervention group had a lower incidence of caries at 2 years compared to those in the control [49], and a recent meta-analysis found that children whose mothers received prenatal oral healthcare had a reduced incidence of early childhood caries up to age 4 [50].

The relationships found in our study between the utilization of preventive medical care and any kind of dental care among mothers and children advance the argument for integration of oral healthcare services within the existing primary healthcare infrastructure, particularly in LMICs. This would enable the transition of the oral healthcare model from one that is emergency-oriented and palliative to one that is truly preventive. Prevention-oriented oral healthcare services that could be delivered within the existing primary healthcare infrastructure and entail minimal training to providers include oral health education with dietary counseling, application of fluoride varnish, oral hygiene instruction, and dispensing of low-cost fluoride toothpaste and toothbrushes. Preventive services such as teeth cleanings and sealants could also be delivered within the primary healthcare infrastructure, but require more training and necessitate an expansion of the oral healthcare workforce. Treatment-oriented services may also be provided within the existing primary healthcare infrastructure. The application of silver diamine fluoride is one therapeutic service that requires few dental tools. Atraumatic restorative technique restorations require more dental equipment, but with proper training could be delivered by mid-level oral healthcare providers. More complicated cases would be referred to dentists and other oral healthcare specialists.

The concept of integration is not new. Since 2002, the WHO's Basic Package of Oral Care (BPOC) has been the framework recommended for integration within primary healthcare and includes urgent oral treatment, atraumatic restorative treatment, and affordable fluoride toothpaste [51]. Nevertheless, for some of the aforementioned reasons, there has been very little improvement in global oral health. In 2021, the WHO renewed its commitment to advancing BPOC [52], and over the past year has been developing a set of oral health strategies to address the remaining challenges. The strategies include integration of oral health as part of Universal Health Coverage agendas, promotion of oral health and prevention of oral disease from individual education to national policies, and development of an oral healthcare workforce that includes mid-level oral healthcare providers, community health workers as well as primary care physicians and nurses [53].

Our study has identified specific opportunities for integration between preventive medical care and oral healthcare, including both prevention- and treatment-oriented services. Our finding that 90% of mothers had received prenatal care with approximately 8 prenatal care visits provides ample opportunities for integration. Similarly, our finding that 95% of children were up-to-date with their immunizations, which entails multiple visits from infancy through preschool-age [54], allows abundant opportunities for integrative services. This would vastly improve oral healthcare access for the 70% of young children in our sample who had never received any kind of dental care. One such case of this integration is the Cambodia Smile program, where trained non-dental primary healthcare workers in immunization clinics provided oral health education and fluoride varnish applications. They found that children in the intervention were 6 times less likely to experience early childhood caries at the age of 2, families experienced improved oral health-related quality of life compared to the control group, and health workers and families found the intervention highly acceptable [55].

This study contributes to the literature by identifying maternal-child oral health concerns and suggesting a specific pathway to improve access to prevention- and treatment-oriented oral healthcare. While our study did not set out to compare differences among countries, the geographically diverse nature of our sample is a notable strength in bringing this global issue to light. The method of convenience sampling was both a strength as it allowed for the inclusion of geographically diverse communities and a study limitation as it can result in selection bias and restrict the generalizability of the findings. Other limitations include survey responses having been affected by recall bias, as mothers were asked about past visits, and information bias if mothers selected answers that they felt the interviewer wanted to hear. As a community-based study, there was inevitably clustering at the community level, for which this exploratory analysis did not account. Finally, given the cross-sectional study design, temporality between preventive medical care and dental care cannot be established. Further research is needed to confirm these findings and test the recommended interventions in a wide range of LMIC settings.

## Conclusion

This study identifies opportunities for integration of oral healthcare services into the existing primary healthcare infrastructure. While prenatal care and child immunization services are widely accessible, general dental care is under-utilized, and a large proportion of mothers and young children are suffering from untreated oral disease and pain. Access to prevention- and treatment-oriented oral healthcare services could be vastly improved by integration into primary healthcare settings, particularly prenatal care and child immunizations services.

## Data Availability Statement

The original datasets analyzed in this study are not publicly available in accordance with participant privacy, informed consent forms that did not include release of the data, and the study's approved IRB protocols. However, the minimal dataset necessary to interpret, replicate and build upon the findings of this study are available from the senior author [KS-G] upon reasonable request.

## Author Contributions

KS-G: conceptualization and data curation. SS and MT: formal statistical analysis. MT: writing—original draft. SS and KS-G: writing—review and editing. SS: visualization and preparation of tables. All authors made significant contribution to the conception or design of the work or to the acquisition, analysis, or interpretation of data. All authors contributed to the article and approved the submitted version.

## Funding

There was no funding for this analysis. Funding for the collection of the original data used in this analysis was supported by small grants from UC Berkeley Professional Development Fund, UC Berkeley Center for Health Research, UC Berkeley Health Research for Action, UC Berkeley Blum Center Big Ideas Fund, UC Berkeley Undergraduate Research Apprenticeship Program, American Academy of Pediatrics I-CATCH, Global Healing, America Nepal Medical Foundation, Jewish Women's Foundation of New York, and donations to the Children's Oral Health and Nutrition Project charitable fund at UC Berkeley. Support for open-access publication fees was provided by the Berkeley Research Impact Initiative (BRII) at the UC Berkeley Library.

## Conflict of Interest

The authors declare that the research was conducted in the absence of any commercial or financial relationships that could be construed as a potential conflict of interest.

## Publisher's Note

All claims expressed in this article are solely those of the authors and do not necessarily represent those of their affiliated organizations, or those of the publisher, the editors and the reviewers. Any product that may be evaluated in this article, or claim that may be made by its manufacturer, is not guaranteed or endorsed by the publisher.
